# Notes on Medical Matters and Medical Men in Paris

**Published:** 1847-04

**Authors:** David W. Yandell

**Affiliations:** Louisville, Ky.


Art. II.—*Notes on Medical Matters and Medical Men in Paris.* By David W. Yandell, M.D., of Louisville, Ky

I spoke in a former communication of the use of some of the preparations of gold by M. Legrand. M. Ricord has been making, at the Hôpital du Midi, some experiments with gold associated with mercury, which, although too few to warrant any definite conclusions, still authorize the hope that it will one day become a highly useful remedy. M. Ricord believes that he has remarked in cases of constitutional syphilis, where he has administered the *chryso-hydrargyrique* pills, the symptoms yield more rapidly than under the mercury alone, without the supervention of any unpleasant accidents either to the mouth or digestive apparatus. He goes so far, indeed, as to insist that the gold, by its association with mercury, acts usefully as a prophylactic against salivation. The *modus operandi* of this amalgam is yet obscurely if at all known. On this subject there is a passage in a work published in 1757, entitled *Cours de Chimie de Lemery*, which, however, the able conductor of the Gazette des Hôpitaux pronounces purely hypothetical. Here is the passage: "Gold is a good remedy for those who have taken too much mercury, for these two metals readily unite with each other, and by this union or amalgamation the mercury is fixed and its progress interrupted. This is well seen in those who have received frictions of mercury, who, if they hold a piece of gold in their mouths for some time, find that it is whitened from the vapor of mercury." The Gazette thus comments upon this: "We have had very lately occasion to treat a patient who had been intensely salivated by a feeble dose of iodide of mercury. A piece of gold well polished was placed in the patient's mouth, where it was allowed to remain during more than half an hour; on being removed, its color was not in the slightest degree changed." Until more ample information is attained upon the subject, the mode in which gold acts, in the cure of disease and in preventing salivation, must remain a matter of conjecture. The amalgam alluded to consists of fifteen parts of mercury to one of gold. The pills, containing from two to three grains of this, are given morning, noon and night.

M. Nélaton communicated, not long since, an extremely curious fact observed by himself in a young epileptic girl, and which, it has been remarked, is without a parallel in the records of medicine. The patient received a blow from a stone on the eye, which contusion was followed by the formation of a traumatic cataract. After the development of the cataract, she became the subject of epileptic fits. Each time that she felt these symptoms, and a few moments before their occurrence, she experienced in the eye a very perceptible pricking, a true *aura epileptica;* and it seemed to her that the general affection, which terminated in the epileptic fit, commenced at the eye. M. Nélaton extracted the crystaline body; the sight was restored to that eye, and the fits ceased. Afterwards, but a long time subsequently, as M. Nélaton has learned, the girl became the subject of mental aberration.

M. Jobert, of St. Louis Hospital, of whom I had occasion to speak in a former letter, makes frequent and successful application, in rheumatism, erysipelas and some other affections, of nitrate of silver in the form of an ointment, varying in strength from four and eight to twelve grammes of nitrate of silver to thirty grammes of lard. (A gramme is 15·444 grains Troy.) The following are a few of the cases in which this ointment was used with the most satisfactory results:

*Observation first.* A man forty-three years old wounded himself in the anterior part of the right leg, with a hatchet. The wound was suppurating at its edges and around them for some lines. The skin presented a manifest erysipelatous blush when M. Jobert saw the patient. The constitutional symptoms were general uneasiness, anorexia, full, hard and frequent pulse. Ordered lemonade, simple dressing to the wound, and application around its whole extent of the ointment of nitrate of silver. The next day the erysipelas had invaded no other region; the local pain and general phenomena were somewhat diminished, but the patient passed a sleepless night, and complained of having felt a lively pain at the internal part of the thigh and groin of the wounded side. The day following, all the symptoms were detected which characterize lymphatic inflammation; the rose-colored bands, sinuous and multiplied at the internal part of the leg, and corresponding to the course of the lymphatic vessels; little erysipelatous wounds corresponding to the more superficial lymphatic ganglia here and there interrupted these lines or bands, and in the same points there was pain, and a cylindrical, fusiform tumefaction, corresponding to the vessels, or to the inflamed ganglia; and finally, the inguinal region of the same side presented many lymphatic ganglia, swollen, and painful upon pressure. M. Jobert ordered all the points which were either red, tumefied or painful, to be anointed with the pommade of nitrate of silver. The next morning, the 15th, the patient said he had passed a better night, and affirmed that the pain which he had felt was sensibly diminished; notwithstanding which, during the days following three other applications of the ointment were made, in order completely to subdue the inflammation and hasten the resolution of the engorged tissues. On the 20th, every complication had disappeared, the wound presented a healthy aspect, the appetite had returned, and the patient was able to be up a little every day. There was a small piece of skin cut by the blow of the hatchet, and left hanging by a slender pedicle, which, notwithstanding the erysipelas, was preserved, and ended by contracting adhesions; but the complete cicatrization of the wound was delayed some weeks longer, when the patient left the hospital perfectly restored, with the inguinal ganglia of the right side no more voluminous or sensible to pressure than those of the left.

*Observation second.* A laborer, æt. forty-eight years, whose right foot had been wounded, presented the following condition: considerable tumefaction, large collection of blood on its dorsal face, and two wounds, one situated at the base of the third toe, the other near the external border of the foot, about two inches in front of the external malleolus. Emollient cataplasms were applied around the foot, which, in addition to venesection, were repeated the next day. On the 13th, the cataplasms were replaced by compresses saturated with camphorated brandy. On the 14th, an erysipelas occupied the dorsal surface of the foot, and had already invaded the inferior part of the leg. A single application of the nitrate of silver ointment sufficed to calm the pain and limit the erysipelas to the parts already mentioned. On the 18th, new symptoms were observed: on the skin of the internal part of the right leg red ribbons, sinuous, very painful to the touch, which gave the sensation of small cylindrical cords, appeared; the ganglia of the groin of the same side were swollen and painful upon pressure. There existed now very evidently in this patient inflammation of the lymphatics and inguinal glands. M. Jobert ordered the ointment of the nitrate of silver to be applied along the whole course of the lymphatic vessels, and upon the ganglia. On the 19th, the pain felt in the groin and thigh had diminished so much that the patient, deprived of sleep the night before, had been able during the last to repose many hours. The pain and swelling of the lymphatics continued rapidly to disappear until completely gone; but the ganglia, although wholly indolent, still preserved a volume a little greater than natural. Dec. 1st, the patient ate and slept well, his general condition very satisfactory. On the 2d, M. Jobert discovered upon the course of the lymphatics three small abscesses, the first situated below the internal malleolus, the second at the internal and inferior part of the leg, the third at the internal and inferior part of the thigh. The introduction of a bistoury was followed by the escape of phlegmonous pus. On the 3d, two other small abscesses were seen, which were similarly opened; one was situated at the internal and superior part of the leg, the other behind the internal condyle of the femur. These five wounds, resulting from the opening of as many little abscesses, were dressed simply. On the 13th, the two wounds on the back of the foot, produced by the accident, were not yet cicatrized; on examination, it was perceived that the skin which separated them was coming away, and that there was already a fistulous communication. M. Jobert introduced a canula, incised the skin, and formed the two wounds into a single one. 19th. For some days the wound on the back of the foot is seen to be cicatrizing; this was favored by touching it with a pencil of nitrate of silver; simple dressing. As to the other small wounds, they are cicatrized; some are still covered with scabs. The lymphatic ganglia of the right groin remain slightly engorged, as is quite manifest to the touch. 27th. The dorsal face of the foot offers a completely continuous and solid cicatrix. The patient has commenced to get up and walk; but the various lesions that he suffered, and especially the repose upon bed that was enjoined, sufficiently explain the slight difficulty which he experiences in movements of the tibio-tarsal articulation of the right side—a difficulty which, without doubt, will disappear after sufficient exercise.

Independently of the interest attached to these two observations under the light of the treatment used, the inflammation of the lymphatics noticed in the subject of the second, presented a remarkable termination. We know that the first effect of inflammation of the lymphatic vessels is the coagulation of the lymph. Generally this lymphatic clot diminishes, is absorbed, and the vessel preserves its calibre. Sometimes the greater part of the fluid of the coagulum is absorbed, the solid part condenses itself in adhesions to the walls of the vessels, and thus obliterates them. At other times again, but more rarely, lymph accumulates at different points, distends the parietes of the vessels, coagulates and adheres there; then these coagula, instead of completely disappearing or of condensing themselves, soften from the centre to the circumference, and furnish pus. Thus result, at different points, and along the course of the lymphatic vessels, true abscesses of small size. We have seen, in the first case, that the patient arrived at the former of these terminations, which of all is the most common and happy; that is to say, that the coagula were absorbed, the vessels preserved their calibre, and the lymph resumed its course. In the second case, on the contrary, the lymphatic inflammation terminated by numerous little abscesses, though the formation of the pus, preceded by adhesions of the clot, was followed by no grave accidents: in fact, pus when thus limited is unable to penetrate the current of the circulation.

*Observation third.* A man, after severe walking and excessive fatigue, was seized with pain in the tibio-tarsal articulation of the right side, and being taken to St. Louis Hospital, his case was pronounced arthritis, with considerable collection of serum about the joint. Anointments with pommade of nitrate of silver were ordered, and after being persisted in for three weeks, the patient left the hospital perfectly cured.

*Observation fourth.* A man entered the same service to be treated for lupus situated at the inferior part of the left leg. During his sojourn at the hospital he was attacked with a violent erysipelas, which rapidly extended over the leg, thigh and buttock of the side corresponding to the lupus. The nitrate of silver was applied by M. Moissenet; the invasion of the erysipelas was arrested, its extent limited, and speedily all the inflammatory symptoms completely disappeared.

*Observation fifth.* A young man was attacked, in March, 1846, with acute rheumatism of the right femoro-tibial articulation, the symptoms of which were so well marked that no doubt could be entertained of the nature of the affection. He was treated energetically by antiphlogistics, and then by sulphate of quinine; notwithstanding which, although the treatment was continued fifteen days, the disease did not terminate by resolution, and some days later the following phenomena were observed: The knee presented considerable tumefaction, and a sensibility so great that the slightest pressure produced the most acute pain; there was flexion of the leg upon the thigh, and the knee exhibited a manifest deformity; the superior extremity of the leg was carried outwards, and the skin was of a violet red. To the touch a sensation was imparted as of foreign bodies contained in the articulation in the midst of a liquid; an exploring puncture proved this to be of a sero-sanguinolent nature.

A white swelling in its commencement was diagnosed, and treated by cauterization and vesicatories, without any amelioration of the symptoms. The nitrate of silver ointment was now used for about fifteen days, when the local symptoms were so greatly diminished that the patient got up and commenced walking without the use of crutches, and continued rapidly to improve until the articulation recovered its form, volume, and healthy movements.

M. Petzold, of Fohrenberg, in an article on the treatment of intermittent fever among very young children, suggests nothing which is not familiar to American physicians, save, probably, his formula for disguising the taste of quinine, which may be of some value:

℞. Clarified honey, 45 grammes;
Sulph. quinine, 75 centigrammes;
Aromatic sulphuric acid, dilute, 4 grammes.

Mix the ingredients thoroughly, of which a teaspoonful is to be given every two hours, shaking it previous to administration. Even this honey will still retain a little bitterness, though children usually take it without any great repugnance. For very young children, the sulphuric acid may be replaced by common water. One or even two teaspoonfuls should be administered every hour during both night and day to children of some size.

*Remedy for Toothache.*—To a hundred grammes of sulphuric ether, in which as large a quantity as possible of camphor has been dissolved, add two or three drops of ammonia; thus is obtained a camphorated ammoniacal ether, which, if applied to carious teeth, immediately relieves the pain. M. Cottereau, who is the author of the preparation, has used it in great numbers of cases with invariable success. The ether evaporates so rapidly that a layer of camphor is left in the dental cavity, which, although too light to incommode as a foreign body, is sufficient to protect the denuded nerve from the air. Besides this, the ammonia acts as a cautery. The solution should be kept in a perfectly closed glass bottle.

Although M. Récamier published the result of some researches on hydatids of the liver as early as 1825, it is in an inaugural dissertation of M. Barrier that we find the complete history, and the establishment, after numerous facts, of the symptomatology, march and termination of this affection. An interesting case of opening of a hydatid cyst into the intestine occurred a short time since in the service of M. Vigla, at the Hôtel Dieu, a rapid and succinct analysis of which may not be out of place. A man 28 years old, of good constitution, lymphatic, has been troubled three or four times in the space of two years with very violent colic, from his description of which it seems much like bilious colic, each attack of which lasted from four to five days, and terminated without any other treatment except rest in bed, diet, and emollient applications to the abdomen. Fifteen days ago he had an attack more violent than any of the preceding, for which he entered the hospital, where the pains augmented in intensity during the early part of his stay, became more acute upon pressure, and were felt especially over the liver; while in the right hypochondriac region, and under the inferior border of the false ribs, a tolerably voluminous, deeply seated and resisting tumor was observed, around which the abdominal parietes, although tense, were smooth and not sensibly elevated or distended by their contents. One morning, after a paroxysm of the most violent pains, the patient was seized with a copious diarrhœa, and almost immediately found himself relieved of all symptoms of disease. The matter which he had passed, preserved and presented at the visit, contained a great quantity of membranous pellicles, opaline, of different sizes, which on an attentive examination were soon recognised as hydatid remains. The next day and the day after, the colics were renewed, and each time followed and relieved by the expulsion of similar matter in large quantities, accompanied by a mucous diarrhœa.

In the space of three days, these accidents were repeated five times, and uniformly terminated in the same way. To-day the patient is in a satisfactory state, complaining of no more pain; the tension of the abdomen has diminished, and the deep-seated tumor, detected at first when the abdominal walls were depressed, is now no longer to be felt.

There had evidently been here a hydatid tumor of the liver, which discharged itself into the digestive tube. According to the light furnished by pathological anatomy and the results of post mortem examinations in analogous cases, we are forced to admit the existence of adhesions which were formed between the walls of the cyst and those of the intestine; which leads to the inquiry, to what part of the intestine were these adhesions formed, and into what part of the digestive tube did the tumor open? It is possible for this to be either in the large or small intestines, and the transverse portion of the colon is but a small distance farther from the liver than the duodenum. As a characteristic symptom, there is nothing to determine the diagnosis, while at the same time there are circumstances which militate in favor of the one opinion rather than the other. In considering the relations of the small intestine with the liver, we see that it was that portion of the intestine which corresponds to the point of the liver towards which the cyst had a tendency to transport itself—that is to say, the anterior and inferior portion. But the circumstance which induces us more readily to believe the opening of the cyst was into the duodenum is, that during the progress of the case entire hydatids were never met with in the stools, but only the remains of hydatid membranes. Now, cysts of this nature contain most frequently a greater or less number of these acephalocysts of every size; and if the cyst had opened into the colon, it would have been almost impossible for some of the least voluminous hydatids not to have passed entire with the discharges; whereas the length of the passage they were obliged to make, if the tumor opened into the duodenum, and the commencement of digestion, mixed with the chyme, the trituration to which the hydatids in the small intestine were necessarily submitted, sufficiently explain the state of disorganization in which they were discharged. The patient is now (December 28th) in a satisfactory condition—a result hardly to have been expected, for, as remarked by M. Barrier, the issue of such cases is most frequently in death. The treatment must of necessity be simply palliative.

M. Briquet, in the treatment of chlorosis, uses sulphate of iron, because of its solubility, in preference to the subcarbonate of the same substance. The following is his method of administering the remedy: Sulphate of iron 1 gramme, distilled water 180 grammes; m. et f. dissolve s. a. Of this the dose is a tablespoonful morning and evening. Each spoonful contains seven centigrammes or about one grain and a half of the salt of iron. The above quantity of the solution is sufficient for daily administration for the space of a week. As to the subcarbonate, much larger doses of that may be administered; two grammes and upwards have been given without any unpleasant effects.

Dr. Guérard has been in the habit of employing with great success, in the chronic diarrhœas which accompany phthisis or enteritis, and which succeed typhoid fever, injections of the following: Common water 1 litre (about a quart English), nitrate of silver 50 centigrammes. Previous to adding the salt to the measure of water, it is easier to dissolve it in a small quantity of distilled water. The patient may without the slightest inconvenience retain or discharge the injection that has been administered, the effect of the argentic salt being the same in either case. M. Guérard has had no occasion to deplore unpleasant accidents from its use.

I promised, in my communication by the steamer of the 5th of January, that at a future time I would complete the history of the case at the Hôtel Dieu, in which ligation of the femoral artery was practised by M. Roux, for a tumor situated at the head of the tibia, soon after which hemorrhage supervened and the man died. The post mortem revelations, which exhibit an error in the diagnosis of the distinguished surgeon, and the correctness of the Gazette des Hôpitaux in its criticisms on the case, are as follows;—previous to giving which, however, I will briefly describe the condition of the patient up to the time of his death:

The operation was performed on the 19th of November, four days after which the first dressings were removed, when the condition of the patient was most satisfactory, the numbness which was so disagreeable at first being almost gone, and the temperature of the member nearly that of the healthy limb. Sixteen days after the ligature was applied a very considerable hemorrhage occurred, which was partially arrested by one of the hospital pupils until M. Roux arrived, who, considering the extreme feebleness of the patient from the loss of blood, and his unwillingness to submit to another operation, as well as for other reasons, contented himself with simply ligaturing the free extremity of the artery a few lines above the point of section, instead of, as might have been done under other circumstances, enlarging the opening in order to apply a ligature beyond the point where the arterial tissues were inflamed. Unhappily, while ligaturing the artery, a large venous trunk was wounded, which circumstance, in conjunction, perhaps, with the evil condition to which the patient had been brought by the loss of blood, developed a phlebitis, a purulent diathesis, and the patient died on the morning of the 11th of December.

At the autopsy, abscesses were found in the spleen and liver, pus existed in the right femoro-tibial articulation, and the tibia itself was so profoundly altered that a quick jerk was sufficient to fracture it. The arterial extremity was still well clasped by the last ligature, and no traces of a second hemorrhage were observable. A section of the tibia perpendicular to its length, made on a level with the superior layers of the bone, showed a healthy osseous tissue; but a longitudinal section revealed a degeneration evidently encephaloid, presenting here and there gelatinous-like points, having destroyed the compact lamellæ of the bone, and constituting the two tumors, or rather the bi-lobed tumor of which I spoke, and in which were felt, before the operation, beats synchronous with the pulsations of the heart. The cartilages at the articulation of the knee had not undergone any alterations of a grave or important character.

This case, evidently beyond the resources of art, affords me an opportunity of giving the four forms of *cancer of the bones* distinguished by M. Nélaton, in doing which, you will not consider me as attempting didactic pathology, but as simply endeavoring, by translating the remarks of one of the latest and most distinguished writers on this subject, to throw light upon a point so difficult and obscure, that a surgeon whose merits no one will question was himself guilty of an error in diagnosis.

1st. In the first form, within the interior itself of the osseous tissue, nuclei of cancerous matter or tissue are found, in cavities which they exactly fill; the tissue of the bone has completely disappeared at the points invaded by the accidental growth, and there is a loss of osseous substance both in its spongy and compact portion, while in the neighborhood of this loss the bone appears to have undergone no alteration, and scarcely more vascularity is remarked than is observed in the normal state of the bone. In proportion as the disease progresses, the cancerous mass becomes developed, and forms a prominence more or less considerable on the surface of the bone, which, I may note, was the case in the patient at the Hôtel Dieu. If the production occupies a long bone and corresponds to its diaphysis, it is not uncommon to see the cancerous growth extend towards the medullary canal, whose membrane offers no resistance, and thus form a tumor which mounts into the interior much higher than its seat or volume would lead one to suppose—a disposition which it is important to bear in mind in cases where it is desirable to resort to amputation of the limb.

2d. In the second form, which is allied more especially to the *osteo-sarcoma* of authors, the tissue of the bone has suffered a profound modification, and generally presents a voluminous tumor, a section of which exposes a great number of cellules, very irregular both in form and dimensions, filled with cancerous matter in different stages of softening, while there seems also to be a tenuity of the osseous tissue in the cellules of which the cancerous matter is deposited.

3d. The third form is that in which a cancerous mass commences in the interior of a bone, develops itself by little and little, and pressing eccentrically the osseous matter, which yields in a slow and gradual manner, becoming thinner and thinner until at length it presents only a very friable shell, in which is contained the cancerous matter; this is the *spina ventosa* of writers. In this variety, when the tumor is sufficiently voluminous, the shell becomes perforated, the cancerous matter escapes by the aperture, forms a prominence under the skin, and goes on like other cancers.

4th. The tumor is attached to the exterior of the bone, covered by periosteum, and has been called *fungous tumor of the periosteum;* though it is easy to see that the osseous tissue is itself altered; in fact, a collection of osseous prolongations extremely fine and flexible are found, which may be compared to hair implanted on the surface of the bone. These points or prolongations are united in the form of a wick or cord, in the intervals and on the surface of which the cancerous matter is deposited.

One sees, adds M. Nélaton, that widely marked differences exist between these forms of cancer of the bones, while at the same time they exhibit the characters proper to cancerous affections. Thus, whatever may be its original form, the tumor constantly increases, softens, ulcerates, almost always reproduces itself after removal, and eventually produces the cancerous cachexia.

An important remark to be made relative to these tumors is, that they never attack the cartilaginous tissue, and that when the cancer has commenced near the articular extremity of a bone, the diarthrodial cartilage is always found untouched, notwithstanding the complete degeneration of the epiphysis which supported it. Whenever the cancerous growth extends into the articulation, it is by a point where the bone is covered only by periosteum and the synovial membrane.

I have very much abridged the anatomical history of this disease of the bones, and will close with a few remarks on the progress and symptomatology of these tumors. They may show themselves without having been preceded or accompanied by pain ; all that occurs, probably, being that at the moment of their development the patient may experience acute but transient pains, perhaps spontaneous, sometimes following fatigue or a sudden movement. Nothing is determined as regards their progress, which at times is slow, and again remarkably rapid. Finally, and this should render one reserved in the exact diagnosis, it must not be forgotten that certain cancers of the bones present beats synchronous with the arterial pulsations—beats which do not consist in a simple rising, but in a true expansive movement, such as is met with in aneurismal tumors. The ear often detects a *bruit de souffle*, less distinct, however, than that of aneurismal tumors; this *bruit de souffle* is not constant, and in the case just related in the service of M. Roux it did not exist. As a differential diagnosis, one will note, when he has been able to observe these tumors from their commencement, that pulsations are felt in aneurisms from the beginning, whereas in cancers they are perceived only at a period when the accidental tissue has become very vascular—that is to say, at an advanced period of their formation.

At the Hospital St. Louis, in July last, there was a case of inguinal hernia reduced spontaneously by the application of a cataplasm. As the case is a singular one, I have thought it sufficiently interesting to translate from the report contained in one of the recent numbers of the Gazette des Hôpitaux:

On the 2d of July, 1846, a man aged 51 years, a shoemaker, of good and robust constitution, entered the wards of M. Malgaigne, having a hernia of the left side, of three years' standing, which had increased gradually and insensibly in volume from the size of a billiard ball to that of a man's fist; it was seated in the groin at first, but finally extended into the scrotum. The hernia returned easily, but escaped upon the least exertion; a bandage was applied, but the patient neglected to wear it constantly, and in the end left it off altogether. Neither of the parents of the patient has had a hernia; one of his brothers has; the patient himself has labored under a cough for four years, to which cause he ascribes the development of the hernia. Tuesday, 30th June, while coughing, the hernia escaped; this time the attempts of the patient to return it were unavailing; he had severe attacks of colic during the nights of Tuesday and Wednesday, and vomited at three different times, at first food, afterwards a greenish colored liquid. Wednesday morning the patient succeeded in effecting a reduction of the tumor; several times during the day his bowels were moved; the condition of things continued so pleasant that he resumed his work without applying the bandage, which was old, worn out and useless. Thursday at 2 o'clock, while going down stairs, the hernia again made its appearance, accompanied by severe pains in the belly, similar to those felt on Tuesday, but without vomiting. All attempts at reduction were ineffectual; the patient had been to stool in the morning; he had breakfasted at 10 o'clock, but was unable to take any more food during the day; at 5, p. m. he entered the hospital, when an inguinal hernia of the left side, which appeared to be strangulated, was observed. Ordered a warm bath, during which taxis was practised by the interne without success, after which a purgative injection was prescribed.

M. Malgaigne being sent for arrived at 10 o'clock in the evening, and detected the following state of things: A pyriform tumor, of the size of the fist, occupying the left side and descending into the scrotum, whose largest extremity was below the smaller or contracted portion looking towards the inguinal ring; the skin which covered it was warm, smooth, shining, much distended, and slightly red in the part occupied by the sack; the tumor was painful on pressure, hard, resisting, and very heavy; perfectly dull; projected forwards so as to displace the penis towards the right side; surface uniform; its pedicle or constricted part seemed like a very hard cord of the size of three fingers, occupying the inguinal canal. The hernia was very distinct from the testicle, which occupied the lower part of the scrotum; it was separated from this organ by a furrow or contraction situated at the distance of two fingers' breadth from the base of the tumor. The abdomen was but slightly painful on pressure, except towards the left half, in the iliac fossa and the flank of the same side. No vomiting or disposition to vomit; patient calm; pulse regular, 76; taxis painful and ineffectual; and the injection, although half an hour had elapsed since its administration, had produced no effect. A large cushion was applied under the hams of the patient, which maintained the thighs flexed upon the pelvis; the tumor was covered by a cataplasm, which was soon followed by marked relief; the patient falling asleep and remaining so until 5 o'clock in the morning, when, awaking, he put his hand upon his groin, and was greatly astonished to find the hernia gone; it had returned entirely through the influence of the cataplasm, which proves the existence of an inflamed hernia.

The reduction was in fact complete; in the forenoon the patient had an abundant stool; and the next day he left the hospital, provided with a bandage. This fact exemplifies in a very striking manner the happy results sometimes obtained, in the most embarrassing cases, by the administration of the simplest remedies, after more active and rational methods have completely failed.

Some weeks ago, in the wards of M. Louis, at the Hôtel Dieu, there was a girl 18 years old, who about a month and a half previously had been delivered of an infant at full term; nothing peculiar was observed during or after the accouchement; the milk fever had been regular in force and duration; on the sixth day after her entrance into the hospital, intense febrile phenomena supervened, accompanied by pains in the hypogastric region, which yielded to the application of some leeches. The fever showed itself again in two days, associated with pains in the abdomen so severe as to draw cries from the patient. These had commenced at midnight the night before; they were greatly increased by pressure, and accompanied by liquid stools, vomiting and extreme pallor of the face; notwithstanding which, M. Louis contented himself with prescribing sinapisms to the lower extremities, and two laudanum injections of fifteen drops each. In half an hour after the visit, in addition to the colics, she suffered with pains commencing at the stomach and extending even to the throat, producing partial suffocation; considerable dyspnœa was experienced, attended by extreme anxiety, and cadaverous hue of the face; the eyes were haggard; movements at times disordered. It was evident that nervous symptoms were present, but arising from a cause which it was at the moment impossible to point out.

M. Louis found the condition of the patient the same the day following, and merely added to the former prescription an antispasmodic. At six o'clock in the evening, a total change had occurred: all the alarming phenomena had disappeared; very little colic remained; respiration sufficiently free; pressure on the belly unaccompanied by pain; no tympanitis; no increase in the volume of the uterus; little fever; and the day after, the patient had appetite for food, desired to get up, and, save the excessive pallor of the face which still continued, her condition was quite satisfactory. The next day and the day after passed without a renewal of the attacks; respiration still unembarrassed; the only phenomena remaining being considerable debility and extreme paleness.

What has been the nature of this apparently formidable but transient affection? The symptoms present indicated an extremely acute peritonitis arising from an intestinal perforation, but there are many reasons which will cause this opinion to be repudiated: In the first place, extreme difficulty of respiration was observed, which is not usually found in these inflammations of the peritoneum; and, what is of still more importance, the termination of the affection, the rapid disappearance of the phenomena, establish conclusively that there was no peritonitis. For the same reasons, there could have been no metritis in the case; added to which it may be remarked that inflammation of the uterus never comes on so suddenly and with so alarming an assemblage of symptoms. Was it an attack of hysteria of a peculiar nature? It is still difficult to admit this. In cases of this description, it is imprudent to give an immediate opinion, as M. Louis showed by reserving his until the following day, when, seeing that the suffocation and pains in the abdomen had entirely ceased, he concluded that all the symptoms were purely nervous, and had been produced by the presence of a clot formed suddenly, from some unknown cause, in one of the ventricles of the heart, which had given rise to considerable embarrassment in the circulation, and afterwards in the general innervation of the economy; the clot having soon become dissolved or absorbed, the crisis had completely passed over, not to be reproduced.

About the same time, phenomena similar to those just detailed were observed in the wards of M. Rostan, in the case of a young woman who was not in the puerperal condition like the former, but who, while enjoying the most perfect health, felt herself so violently oppressed, that, to use her own words, "she thought she could not live an hour." At the same time that she was a prey to this sense of suffocation, the most distressing pains in the abdomen and chest were experienced, those in the precordial region being most severe. The first attacks came on three weeks ago, since which time the same symptoms have repeatedly shown themselves, and during the intervals—that is, during the moments of calm—the pulse is so small and threadlike that it can hardly be felt at the wrists. The abdominal and precordial pains persist, though less severe; auscultation of the chest reveals a normal vesicular respiration, while the pulsations of the heart are dull, deep, difficult to distinguish clearly, joined to a *bruit de souffle* which predominates over the respiratory sound; marked dullness from the third to the seventh rib upon percussion, except which, all the functions are properly executed; there being no marked thirst, cephalalgia, heat of the skin, or sweatings: appetite and digestion good; stools healthy; the menstrual flow has not appeared for three months.

Concerning this patient M. Rostan made the following observations: It is clear that this was an acute malady, seated, where? All the indications show that it was in the heart or its envelops; but was it a pericarditis, or an inflammation of the heart itself? The professor is inclined to the latter opinion; but it is nevertheless proper to remark, that carditis alone does not explain all the phenomena observed; there must have been something else, which is found in inflammation of the internal membrane, designated by Broussais *internal carditis*, and by late writers *endocarditis*, which induced the formation of sanguine clots and their retention in the cavities of the heart. The enormous extent of the dullness in the precordial region would lead to the belief that there was also, perhaps, pericarditis with serous effusion. Following this diagnosis, the treatment should be antiphlogistic, which in fact was the case; the patient has been twice bled, and cups have been applied over the region of the heart; and under this plan a prompt and sensible amelioration has resulted.

In cases of this description, the advantages of sanguine depletion are numerous. When the pulse is of that reduced size already alluded to, bleeding develops it by dissipating the inflammation; while another effect is the diminution of the plasticity of the blood, in consequence of which the tendency to the formation of clots is greatly lessened, and if already formed, it diminishes their chances to augment in volume.

It is to be remarked that although these two cases are not without certain analogies, still there is a very great difference between them. In that of Louis, the brief duration of the symptoms forbids the supposition, that the clot could have formed in the heart in consequence of an inflammation of the substance of the organ. What then could have been the determining cause of this coagulation of the blood? Evidently it is beyond our power to say in a positive manner, though, since it is reduced to hypothesis, there is nothing to hinder the search for the cause of the phenomena in the nervous system.

If nervous congestions are admitted to be possible, and to be capable of being the cause of real disorders, who will oppose, in a case of this kind, our ascribing to a disturbance of the general innervation a disorder, of which the occasional cause escapes our notice, viz., the formation of a clot in one of the ventricles of the heart?

I pass to the third case, the subject of which was a woman aged 18 years, a milliner, who had been constipated for some days, without either much pain in the abdomen, sensibility on pressure, rumbling of the intestines—in a word, without anything which indicated a typhoid state. The respiratory murmur was normal throughout the lung, save at the right side, where, in a limited point, a light *bruit de souffle*, perhaps physiological, was detected; the sensibility of the abdomen was obtuse, which is explained by the obstinate constipation, dating now eight days, at which time her menstrual discharge being present, was suddenly checked after exposure to cold, which is the only cause the patient is able to assign for her affection; intense cephalalgia is the only phenomenon on the part of the nervous system; dullness of the heart in its normal limits; pulsations strong; the first sound accompanied by a distinct *souffle;* pulse from 104 to 108. Venesection to four hundred grammes.

The next day M. Rostan saw the patient, and adopting the ideas of his *chef de clinique*, (who had ordered the first bleeding,) relative to a probable inflammation of the heart, prescribed another venesection, which furnished a clot with a firm, thick, buffy coat; cups were applied in the evening; the *bruit de souffle* had diminished perceptibly under the influence of the general and local depletion, each of which had been practised three times in four days, and at the end of five days no traces of the *bruit de souffle* remained. Rostan thinks there was in this case clearly an inflammation of the internal membrane of the heart, perhaps of the tissue itself of the organ, supervening without any appreciable cause, and neither accompanied, as in the preceding cases, by the formation of clots, nor by that reduction of the pulse so remarkable in the second, and so characteristic of it.

A case of inflammation of the subcutaneous cellular tissue of the right leg occurred in the wards of M. Gerdy, at La Charité Hospital, a few days ago, which was successfully treated by simply elevating the member, a brief outline of which I believe will be found interesting. The patient was a young man of a good constitution, who presented, at the time of his admission into the hospital, a commencing phlegmon which as yet offered no appreciable fluctuation; the skin was red and hot; the tumor was quite prominent, and, without doubt, was produced by an inflammation of the subcutaneous cellular tissue; already it involved a large portion of the right leg. M. Gerdy placed the affected limb in such a position that the foot was considerably elevated above the plane of the pelvis, and under this treatment alone in a short time the inflammatory action diminished in intensity, the redness and heat became less, resolution was effected rapidly, and a complete recovery resulted.

Dr. Rayer, of whom I spoke in a previous letter, has been in the habit of using, with most satisfactory results, the oil of croton tiglium in frictions on the anterior surface of the thoracic cavity, in those persons who were laboring under pulmonary tuberculization. According to this able practitioner, twenty-four drops of the oil may be used for each friction with impunity. It is used by pouring a little at a time on the chest, and then rubbing it with the naked palm of the hand; this is followed, as is well known, by the development of pimples or buttons on the breast, which the hand escapes, owing, perhaps, to the absence of follicles and the greater thickness of the epidermis on its palmar portion. The employment of the croton oil in this way and in this quantity affords notable relief to the dyspnœa, the nocturnal agitation, and the fever which so cruelly torment patients of this kind; and it is to be regretted that the high price of the article places it beyond the reach of the indigent, and must seriously limit its application in such affections.

Here I may mention that a medical friend from New York, now in Paris, has assured me that, during the last four or five years, he has applied the croton oil in frictions to the anterior and superior surface of his chest whenever he has been troubled with a cold which affected especially the bronchia, and that it has always afforded relief within twenty-four hours. He thinks the pustules form more readily if the part has previously been washed well with warm water, and afterwards rubbed with a rag saturated with spirits of camphor. Previously to commencing frictions with the oil, which he usually does in the evening before going to bed, he takes care to place a handkerchief between the thorax and his under-shirt, in order to prevent the latter from becoming soiled when the pustules break.

Dr. Rognetta says that he cannot too highly recommend in smallpox the application of mercurial ointment, solidified by the aid of starch or flour, which he renews once or twice during the day. A thick layer of the pommade is spread with the finger on the forehead, cheeks, lips, eyes, nose, ears, etc. The buttons are aborted in these regions, the eyes are perfectly preserved; the face, nose and lips do not experience that horrible and acutely painful swelling which belongs to confluent variola, and the patients feel themselves refreshed by each application of the mercurial ointment; in a word, it is positive that this agent is really a precious remedy, not only as a preservative against cicatrices, but, what is still more useful, as a true safeguard to the eyes, and a preventive of the distressing pains of which the patients complain so much. These results have been proved by M. Rognetta in the wards of M. Briquet at La Charité; besides which he advises bloodletting in adults in smallpox, adding that he has seen venesection practised in the hospitals in Italy, especially at the commencement of the affection, not only once, but four, five, and even six times, with the happiest effects. If the termination of the disease is fatal, it is because of the disease itself, and not the bleeding. He treats the fear of purulent absorption as a mere vagary, and considers that abstraction of blood cannot but facilitate the march of the eruption. Of course among children the use of the lancet is inadmissible.

M. Pelouze, at a late meeting of the Academy of Sciences, made some remarks concerning a letter of Prof. Liebig relative to certain new researches in animal chemistry. M. Liebig, after washing large quantities of hashed meat and collecting the water used in the operation, has detected in the muscular flesh the constant presence of an acid which was first discovered in milk, and hence called lactic acid; this acid thus spread through the flesh is separated from the blood only by fine and permeable membranes. Prof. Liebig finds in the approximation of this acid and the alkaline principle of the blood the previous cause of the electrical currents discovered in the muscles by M. Matteucci. While performing these delicate manipulations, Liebig has met with a substance, the knowledge of which is due to M. Chevreul, and which he designates by the name of *creantine.* It is found in very small quantities, but its existence is constant. It has been detected in the flesh of the ox, calf, sheep, hog, horse, hare, fowl, and pike fish. Is it to the creantine that the soup of meat owes the nutritive qualities which are possessed by soups obtained from no other parts of the animal organization but the muscles. Liebig considers that it is the creantine which plays the most important part in the living economy.

M. Pellerin has just communicated some new and striking views respecting sea-sickness. He maintains that the opinions that attribute this affection to a congestion of the brain, and to a disturbance in the abdominal viscera produced by the motion of the vessel, are alike groundless. According to M. Pellerin, the heaving and rolling of a vessel produce a derangement in the circulation, which causes sea-sickness; the result of this derangement is not, as is stated by Wollaston, a congestion of the brain, but, on the contrary, this organ is deprived of that amount of blood necessary for its stimulation. The sensations of sea-sickness are much like those experienced when a patient has been bled to the point of syncope. In support of his view of the pathology of this affection, M. Pellerin mentions, what every one who has been sea-sick knows, that its violence is greatly lessened by assuming the horizontal position, a circumstance which, I need hardly say, is witnessed also in cases of syncope. In pregnant women, the uterus requiring a larger amount of blood than under other circumstances, the brain is deprived of a portion of that which it ordinarily receives, and thus nausea is produced, between which and the sensations of sea-sickness Mons. P. draws an analogy, strongly supported by the fact, that the nausea of pregnancy is rare while the female is lying in bed, but is frequently felt when she gets up. Adopting this as the correct opinion in relation to sea-sickness, the remedies should be everything calculated to determine blood to the head, such as belts around the body, etc. He further suggests that persons who are troubled with a tendency to a flow of blood to the head should try sea-sickness as a remedy; and certainly, if he is right in his theory, his suggestion is not unsupported by reason.

Knowing that the journals, medical and political, abound in accounts of the use of vapor of ether, I content myself with simply saying, that the evidences in its favor are so irresistible, that no observer can withstand the conviction of the powers attributed to it by American and English surgeons; and that where there was at first the most unscientific opposition to its use, there is now the utmost eagerness to promulgate its, I may say, blessed properties. From a Paris English paper I clip the following extract, containing the statement of M. Gerdy, made before the Academy of Sciences at its last meeting, relative to the effects of the inhalation upon himself:

After stating that he inhaled the ether through the tube of a bottle containing about three pints, he says: "The irritation which I felt at first in the throat made me cough; but being resolved to resist, I soon triumphed over this little obstacle. The irritation and cough gave way as the inhalation continued. I next experienced a numbness of the head, attended with heat, as if the vapor of alcoholic and intoxicating liquor was mounting to the brain. This numbness extended rapidly first to the feet, and then to the legs and arms, and next to the loins, and increased rapidly with each inspiration. In the organs of sensation it was attended with an agreeable feeling of heat, and of a vibration similar to that which we experience in touching a vibrating body, such, for instance, as a large bell when struck by its hammer. When these two sensations reached their maximum, I experienced an impression both agreeable and voluptuous, like that of intoxication. It is the numbness of which I speak that diminishes the pain in operations. My sight was not sensibly benumbed; the hearing was more so, and it became more and more feeble as the intoxication increased. I convinced myself, however, that the smell, the taste, and the touch, properly speaking, were not paralyzed by the general numbness which came over me; but my eyelids became heavy, and I felt a desire to give myself up to the charms of my intoxication."

Liston and Ferguson, of London, have employed the vapor preparatory to their operations with most decided success, several of their patients having been wholly unconscious of pain.

*A correction.*—In my last letter (pages 229, '30, March number of this Journal.) I was betrayed into an error by an incorrect report of Béhrier's lecture on *smallpox.* I find the error corrected in another report of the lecture. For "variola," "varioloid" should be substituted, and the sentence will then read, You very rarely find umbilical pustules in varioloid.

Paris, January 26, 1847.

